# Guter Ausgang nach OHCA trotz infauster Prognose und Verzicht auf eCPR

**DOI:** 10.1007/s00101-026-01672-4

**Published:** 2026-04-02

**Authors:** Susanne Waitz, Christine Kleindorfer, Liudmila Belevskaia, Alexander Leibold, Peter Paul Ellmauer, Jan Dembianny, Dirk Lunz, Bernhard Graf, Dominic Barthelmes

**Affiliations:** 1 Klinik für Anästhesiologie, Universitätsklinikum Regensburg, Franz-Josef-Strauß-Allee 11, 93053 Regensburg, Deutschland; 2 Barmherzige Brüder Klinik St. Hedwig, Steinmetzstraße 1–3, 93049 Regensburg, Deutschland; 3 Klinik für Herz‑, Thorax- und herznahe Gefäßchirurgie – Fachbereich Kardiotechnik, Universitätsklinikum Regensburg, Franz-Josef-Strauß-Allee 11, 93053 Regensburg, Deutschland

## Anamnese

Ein 50-jähriger Mann erleidet um 18:42 Uhr zu Hause plötzlich einen beobachteten Kollaps und wird unmittelbar bewusstlos. Die Tochter alarmiert die Rettungsleitstelle und beginnt unter telefonischer Anleitung bei fehlenden Lebenszeichen mit der Herzdruckmassage. Es werden ein Rettungswagen (RTW), ein Notarzteinsatzfahrzeug (NEF) und ein Helfer vor Ort (HVO – speziell ausgebildete, ehrenamtliche Einsatzkraft, die bei Notfällen vor dem Rettungsdienst am Einsatzort eintrifft und erste Hilfe leistet) alarmiert.

## Einsatzverlauf

Der HVO übernimmt um 18:52 Uhr nach Beginn der Laienreanimation den *Basic Life Support *(BLS). Mit dem Eintreffen von RTW (18:57 Uhr) und Notärztin (19:01 Uhr) startet der *Advanced Cardiac Life Support* (ACLS). Anfangs liegt Kammerflimmern (KF) vor, das durch Defibrillation (18:59 Uhr) kurzzeitig in einen Spontankreislauf (ROSC) übergeht. Zwei Minuten später zeigt sich eine pulslose elektrische Aktivität (PEA). Ab 19:01 Uhr übernimmt die Notärztin (Fachärztin für Anästhesiologie und Notfallmedizin) die Maskenbeatmung mit 100 %igem Sauerstoff. Die Intubation scheitert aufgrund eines schwierigen Atemwegs (C/L III). Es wird das *ExtraCorporeal-Life-Support*(ECLS)-Team alarmiert, das zudem ein Videolaryngoskop mitführt. Auf einen erneuten Intubationsversuch wird daher zunächst verzichtet. Weitere Atemwegsinterventionen, einschließlich supraglottischer Atemwegshilfen, werden bewusst unterlassen, da unter Reanimationsbedingungen mit erhöhten Beatmungsdrücken kein klarer Vorteil gegenüber der suffizienten Maskenbeatmung besteht und relevante Leckagen zu erwarten waren. Die absehbar zeitnahe Ankunft des ECMO-Teams mit erweitertem Atemweg-Equipment (einschließlich Videolaryngoskop) wird zusätzlich in die Entscheidungsfindung einbezogen.

Es erfolgt die problemlose Anlage eines venösen Zugangs in die *V. jugularis externa*. Um 19:25 Uhr trifft das ECLS-Team (Facharzt für Anästhesiologie und Notfallmedizin, Kardiotechniker) ein. Im weiteren Verlauf besteht eine persistierende Reanimationspflichtigkeit mit wechselnden Rhythmen zwischen KF und PEA. Es sind 6 Defibrillationen durchgeführt und nach ACLS-Schema Adrenalin und Amiodaron verabreicht worden. Mit Hilfe des Videolaryngoskops und unter Bougie-Assistenz gelingt die Intubation im zweiten Versuch. Um 19:30 erfolgt die Evaluation zum Beginn einer *extracorporeal Cardiopulmonary Rescuscitation* (eCPR) auf Basis folgender Befunde:

- Patient im Hausflur, unzureichende Lichtverhältnisse,
- CPR-Dauer mittlerweile 43 min, initialer Rhythmus KF,
- laufender ACLS, mit Rhythmus-Downgrade von KF zu PEA (wechselnd),
- mutmaßlich prolongierte Hypoxie bei schwierigem Atemweg,
- ausgeprägte Zyanose im Bereich des Kopfes und der oberen Thoraxapertur,
- niemals *signs of life*,
- etCO_2_ initial nach erfolgreicher Intubation 14 mm Hg,
- rSO_2_ 15 % im frontalen Kortex (INVOS^TM^ 7100 System (Medtronic, Minneapolis, MN, USA)),
- apparative Pupillometrie beidseits weit und lichtstarr (NPi Wert 0, NPi®-300 Pupillometer, NeurOptics),

## Entscheidungsfindung eCPR

Nach Sicherung des Atemwegs wird die Indikation zur eCPR-Initiierung gemeinsam zwischen Notärztin, ECLS-Team sowie zusätzlich aufgrund des jungen Patientenalters, der prolongierten Reanimationsdauer sowie den gleichzeitig fehlenden anamnestischen Hinweisen auf relevante Vorerkrankungen dem Hintergrunddienst des ECLS-Zentrums (Facharzt für Anästhesiologie, Kardioanästhesie, spezielle Intensivmedizin, Notfallmedizin) um 19:30 Uhr diskutiert. In Zusammenschau der oben genannten Befunde wird sich zeitnah im Konsens gegen eine eCPR entschieden. Parallel erfolgt ein Gespräch mit der Angehörigen, während die Reanimationsmaßnahmen zunächst fortgeführt werden. Bei persistierender pulsloser elektrischer Aktivität (PEA) ist die Beendigung der Reanimation vorgesehen, während beim Auftreten von Kammerflimmern Defibrillation und weiterführende Therapiemaßnahmen konsequent fortgesetzt werden.

## Weiterer präklinischer Verlauf

Unter maschineller Beatmung und erneuter Defibrillation sowie Suprareningabe kommt es noch vor Abbruch der Maßnahmen nach 83 min Reanimationsdauer um 20:05 Uhr zu einem ROSC. Der Patient wird analgosediert und erhält niedrigdosiertes Noradrenalin (0,02 µg/kgKG und min). Das EKG zeigt einen Sinusrhythmus mit einem kombinierten Rechtsschenkelblock und linksanteriorem Hemiblock ohne ST-Hebungen. Der Patient wird unter kontrollierter Beatmung bodengebunden in ein Krankenhaus der Versorgungsstufe I transportiert.

## Innerklinischer Verlauf

In Rahmen der Schockraumversorgung (Tab. 1) ist der Patient kardiorespiratorisch stabil. Unter Analgosedierung wird der Patient auf die Intensivstation gefahren, wo er bei Eintreffen eine katecholaminpflichtige Kreislaufinsuffizienz sowie erneut Kammerflimmern entwickelt. Nach 5‑minütigem ACLS mit 2 Defibrillationen ergibt sich ein erneuter stabiler ROSC. Das EKG bleibt unverändert.

**Tab. 1:** Befunde Schockraumversorgung

Diagnostik	Befund
Pupillenweite	Beidseits mittelweit, lichtreagibel
Blutdruck	Stabil, MAP 65–85 mm Hg
EKG	Sinusrhythmus mit bifaszikulärem Block, Frequenz 90/min, pathologische Q‑Zacke in II, III, aVF; T‑Negativierung V_3_–V_6_
Beatmung	BiPAP/ASB, F_I_O_2_ 0,5, P_peak/mean_ 20/10 mbar
Herzecho (orientierend)	Höhergradig reduzierte EF ohne H. a. regionale Wandbewegungsstörungen, höhergradige Stenosen der Aorten‑/Mitralklappe oder schwerer RV-Dysfunktion
cCT/CT	Reanimationsfolgen: Sternum- und rechtsseitige Rippenfraktur (4./5.), V. a. Aspirationspneumonie. Ausschluss zerebraler Akutpathologie und Lungenarterienembolie
BGA	Lactat 3,44 mmol/l, p_a_CO_2_ 56 mm Hg, p_a_O_2_ 92 mm Hg, K^+^ 3,5 mmol/l, Hb 6,2 g/dl, pH-Wert 7,21, Glucose 172 mg/dl
Medikation	Noradrenalin, Sufentanil, Midazolam
Nun bekannte Vorerkrankungen	2‑Gefäß KHK mit Z. n. 2‑mal DES 2022
	Kompletter Rechtsschenkelblock 2022
	Z. n. linkszerebraler Ischämie bei Verschluss der A. cerebri media, keine Residuen
	Ischämische Kardiomyopathie mit mittelgradig eingeschränkter linksventrikulärer Funktion (EF 35 %–40 %)

Aufgrund bereits kurz nach Aufnahme auf die Intensivstation beobachteter gezielter Reaktionen wird frühzeitig ein Aufwachversuch durchgeführt, wodurch eine prolongierte Sedierung vermieden wurde. Nach Beendigung der Katecholamintherapie um 01:30 Uhr erfolgt die komplikationslose Extubation.

Neurologisch zeigt sich lediglich eine retrograde Amnesie. Bei Ausbildung pathologischer Q‑Zacken sowie einer positiven Troponindynamik wird der Patient bei fehlender Katheterdiagnostik der erstversorgenden Klinik noch in der Nacht in ein Zentrum mit Herzkatheterlabor (Versorgungsstufe III) verlegt, wo ein RIVA-Gefäßverschluss interventionell rekanalisiert wird. Bei nächtlichen Sinusbradykardien sowie einer eingeschränkten linksventrikulären Ejektionsfraktion (EF) von 35 % wird im Verlauf ein DDD-AICD Schrittmacher implantiert. Ob das Kammerflimmern Folge einer akuten Plaqueruptur oder Folge einer malignen Herzrhythmusstörung bei schwerer KHK mit chronisch verschlossener RIVA war, ist abschließend nicht zu klären.

Der Patient wird an Tag 14 neurologisch uneingeschränkt und in gutem Allgemeinzustand entlassen.

## Diskussion

Dieser Fall verdeutlicht die wesentliche Schwierigkeit der präklinischen Entscheidungsfindung zur eCPR bei außerklinischem Herz-Kreislauf-Stillstand (OHCA): Hat die bisherige Reanimation überhaupt die Voraussetzungen für eine erfolgreiche eCPR geschaffen?

Sieht man von Organerhalt zur Transplantation ab [1], ist das Therapieziel der eCPR ein neurologisch günstiges Outcome (mRS 0–3 bzw. CPC I–II) [2]. Dies geht einher mit einer frühen und qualitativ hochwertigen Reanimation zur Sicherstellung einer ausreichenden Perfusion des Gehirns [3]. In dem geschilderten Fall war beim Eintreffen des ECLS-Teams jedoch unklar, ob die Reanimationsmaßnahmen tatsächlich effektiv waren, oder ob bereits ein neurologischer Schaden eingetreten ist. Bisher kann die Qualität der Reanimation insbesondere im präklinischen Bereich, wo aufwendigere Verfahren wie die transösophageale Echokardiographie [4] oder invasive Messung von Blutdrücken [5] bisher nicht routinemäßig verwendet werden, nur indirekt beurteilt werden. Hierfür wurden Prädiktoren zur Nutzen-Risiko-Abwägung entwickelt [6, 7], um Patienten mit sehr geringer Überlebenswahrscheinlichkeit frühzeitig zu identifizieren [8–10]. Im geschilderten Fall sind mehrere Kriterien erfüllt, die für einen unzureichenden Reanimationserfolg sprechen: eine Low-Flow-Zeit > 20 min, ein rSO_2_ < 20 % sowie bilaterale Lichtstarre der Pupillen. werden. In Kohorten für präklinische eCPR findet sich in dieser Konstellation selbst bei zeitnaher ECMO-Implantation nur eine sehr geringe Wahrscheinlichkeit für ein gutes neurologisches Outcome (< 6,4 %) [9]. Mit zunehmender Reanimationsdauer sinkt die Wahrscheinlichkeit eines neurologisch intakten Überlebens deutlich. Bereits oberhalb von 20 min ist sie gering [11], und *Low-Flow*-Zeiten > 45 min sind mit einer signifikant erhöhten Mortalität assoziiert [12]. Klinische Lebenszeichen unter Reanimation, die prognostisch günstig sind, wurden nicht beobachtet; jedoch ist nicht auszuschließen, dass Zeichen wie Spontanatmung oder -bewegung unter den Einsatzumständen (begrenzte Lichtverhältnisse und beengte Räumlichkeit) übersehen wurden. Das interdisziplinäre Team hat in Zusammenschau der Befunde eine sehr geringe Wahrscheinlichkeit für ein günstiges neurologisches Outcome angenommen und sich zur Vermeidung der bei eCPR vermutlich erhöht beobachteten Rate an Übertherapie [13] konsensuell gegen eCPR entschieden. Umso bemerkenswerter ist der im weiteren Verlauf erreichte ROSC mit unerwartet gutem neurologischem Ergebnis. Möglicherweise – und vielleicht auch im Sinne einer glücklichen Fügung – hat gerade der Verzicht auf die va-ECMO-Anlage, trotz *ex post* erkennbarer Fehleinschätzung der Neuroprognose, die schnelle Rekonvaleszenz mit *Restitutio ad integrum* begünstigt, da die mit eCPR verbundene Morbidität und Mortalität [14–16] vermieden wurde.

Fallberichte mit unerwartet guten Ausgängen trotz prolongierter Reanimationsdauer [17] oder fehlender Pupillomotorik [18] bestätigen unsere Schlussfolgerung aus der hier präsentierten Kasuistik, dass die Qualität der Reanimation entscheidend ist, aber bisher nur unzureichend beurteilt werden kann. Gerade im Hinblick auf die Indikationsstellung zur eCPR müssen die etablierten Prädiktoren mit Bedacht verwendet werden, und jede eCPR-Evaluation wird weiterhin eine interdisziplinäre Einzelfallentscheidung mit hoher Unsicherheit bleiben.

## Fazit für die Praxis

- Der Fall zeigt, dass trotz prolongierter Reanimation ein gutes neurologisches Outcome möglich ist und etablierte Prädiktoren zur eCPR-Indikationsstellung nur eingeschränkt verlässlich sind.
- Da die Reanimationsqualität präklinisch schwer zu beurteilen bleibt, müssen Entscheidungen weiterhin individuell und interdisziplinär erfolgen, um Patienten mit realem Nutzen durch eine eCPR zu identifizieren und zugleich unnötige belastende Maßnahmen zu vermeiden.
